# Common Variants in *MAGI2* Gene Are Associated with Increased Risk for Cognitive Impairment in Schizophrenic Patients

**DOI:** 10.1371/journal.pone.0036836

**Published:** 2012-05-23

**Authors:** Takayoshi Koide, Masahiro Banno, Branko Aleksic, Saori Yamashita, Tsutomu Kikuchi, Kunihiro Kohmura, Yasunori Adachi, Naoko Kawano, Itaru Kushima, Yukako Nakamura, Takashi Okada, Masashi Ikeda, Kazutaka Ohi, Yuka Yasuda, Ryota Hashimoto, Toshiya Inada, Hiroshi Ujike, Tetsuya Iidaka, Michio Suzuki, Masatoshi Takeda, Nakao Iwata, Norio Ozaki

**Affiliations:** 1 Department of Psychiatry, Nagoya University Graduate School of Medicine, Nagoya, Japan; 2 Department of Psychiatry, Fujita Health University School of Medicine, Toyoake, Japan; 3 Department of Psychiatry, Osaka University Graduate School of Medicine, Osaka, Japan; 4 Molecular Research Center for Children’s Mental Development, United Graduate School of Child Development, Osaka University, Kanazawa University, and Hamamatsu University Graduate School of Medicine, Osaka, Japan; 5 Department of Psychiatry, Seiwa Hospital, Institute of Neuropsychiatry, Tokyo, Japan; 6 Department of Neuropsychiatry, Okayama University Graduate School of Medicine and Dentistry, Okayama, Japan; 7 Department of Neuropsychiatry, University of Toyama Graduate School of Medicine and Pharmaceutical Sciences, Toyama, Japan; 8 CREST, Japan Science and Technology Agency, Tokyo, Japan; National Taiwan University, Taiwan

## Abstract

Schizophrenia is a complex psychiatric disorder characterized by positive symptoms, negative symptoms, and cognitive impairment. *MAGI2,* a relatively large gene (∼1.5 Mbps) that maps to chromosome 7q21, is involved in recruitment of neurotransmitter receptors such as AMPA- and NMDA-type glutamate receptors. A genetic association study designed to evaluate the association between *MAGI2* and cognitive performance or schizophrenia has not been conducted. In this case-control study, we examined the relationship of single nucleotide polymorphism (SNP) variations in *MAGI2* and risk for schizophrenia in a large Japanese sample and explored the potential relationships between variations in *MAGI2* and aspects of human cognitive function related to glutamate activity. Based on the result of first schizophrenia genome-wide association study in a Japanese population (JGWAS), we selected four independent SNPs and performed an association study using a large independent Japanese sample set (cases 1624, controls 1621). Wisconsin Card Sorting Test (WCST) was used to evaluate executive function in 114 cases and 91 controls. We found suggestive evidence for genetic association of common SNPs within *MAGI2* locus and schizophrenia in Japanese population. Furthermore in terms of association between *MAGI2* and cognitive performance, we observed that genotype effect of rs2190665 on WCST score was significant (p = 0.034) and rs4729938 trended toward significance (p = 0.08). In conclusion, although we could not detect strong genetic evidence for association of common variants in *MAGI2* and increased schizophrenia risk in a Japanese population, these SNPs may increase risk of cognitive impairment in schizophrenic patients.

## Introduction

Schizophrenia is a complex psychiatric disorder, affecting approximately 1% of the general population. It is characterized by positive symptoms, negative symptoms, and cognitive impairment. The heritability of schizophrenia is estimated to be 64% [1]. Although genes relevant for schizophrenia or variants that may modulate risk for the disease have been identified using both linkage- and candidate-based or whole genome association studies, the genetic basis of schizophrenia is still unclear [2], [3], [4], [5].

Considerable evidence supports a relationship between cognitive impairment and functional outcome in schizophrenia [6]. Cognitive impairment is considered a core feature of schizophrenia that includes problems is processing speed, attention/vigilance, working memory, verbal learning, visual learning, problem solving, and social cognition. The molecular mechanisms responsible for cognitive deficits in schizophrenia are to some extent related to impaired synaptic plasticity [7]; many of the key components involved are synaptic scaffolding molecules of the membrane-associated guanylate kinase (MAGUK) family, such as PSD-95 and PSD-93 [8]. Closely related to the MAGUK family of scaffolding molecules are the inverted MAGUKs (MAGIs). Mammalian MAGIs are found at both synapses and epithelial junctions [9]. However only MAGI2 (also called synaptic scaffolding molecule, or S-SCAM) is brain-specific [10] and has been shown to interact with NMDA receptors at excitatory synapses [9].

Human *MAGI2* is relatively large gene (∼1.5 Mbps) that maps to chromosome 7q21. Interestingly, recent copy number variation analyses have identified deletions in gene coding *MAGI2* in schizophrenia [11]. *MAGI2* is also interesting from a biological point of view. One study showed that MAGI2 is involved in recruitment of neurotransmitter receptors such as AMPA- and NMDA-type glutamate receptors [12]. MAGI2 is present at glutamatergic synapses, where it interacts with NMDA receptors, neuroligin1, and β-catenin and at GABAergic synapses, it has been reported that MAGI2 can interact with β-dystroglycan and neuroligin2, suggesting that this scaffold molecule may provide a link between the DGC and the neurexin–neuroligin adhesion system [13]. Moreover, in *Caenorhabditis elegans*, mutation in magi-1 (a close homolog of mammalian MAGI2 proteins) leads to deficits in neuron-specific regulation of associative learning and memory [14], while complete deletion of magi-1 is associated with the experience-dependent regulation of subunit-specific AMPAR trafficking and behavioral plasticity as well as defects in long-term memory acquisition in vivo [15].

To the best of our knowledge, no genetic association study specifically designed to evaluate the association between *MAGI2* and cognitive performance or schizophrenia has been conducted. However, several weak association signals (p<0.05) within the *MAGI2* locus were detected in the first genome-wide association study of schizophrenia conducted in a Japanese population (JGWAS) [16]. It is of note that in this JGWAS, no genome-wide evidence for an association was detected, and the non-genome-wide level of statistical significance should be interpreted with caution. However, due to the relatively small sample size, a type II error (false-negative result) cannot be excluded [17], especially in cases of small odds ratios (OR), which are expected for common single nucleotide polymorphisms (SNPs) associated with schizophrenia.

In this study, we examined the relationship of SNP variations in *MAGI2* and the risk for schizophrenia in a large Japanese case-control sample and also explored potential relationships between variations in *MAGI2* and aspects of human cognitive function related to glutamate activity. We reasoned that if *MAGI2* increases the risk for psychosis by virtue of its putative role as an indirect modulator of NMDA neurotransmission, it should also impact cognitive function associated with NMDA signaling, which has also been associated with psychosis.

## Results

### Association Study

In the confirmation sample set, no SNPs showed a p value less than 0.05. HWE p values in controls were not significant. In the joint analysis, two SNPs showed p values less than 0.05 (rs2190665: p = 0.0033, odds ratio of minor allele 0.88, rs4729938: p = 0.027, odds ratio of minor allele 1.11) (Table 1). The calculated threshold by the SNPSpD was at p = 0.125 (Supplementary Methods S1) [18].

**Table 1:** Results of association study and joint analysis.

Number	SNP	Confirmation sample (n = 3245)			OR^2^	L95	U95	HWEp^3^	Confirmation sample + JGWAS (n = 4353)				
		Cases	Controls	P-value^1^					P-value^1^	OR^2^	L95	U95	P_BD^4^
1	rs2190665	0.39	0.42	0.06	0.91	0.82	1.01	0.79	**0.0033**	0.88	0.80	0.96	0.22
2	rs10260177	0.04	0.04	0.39	1.12	0.87	1.44	0.49	0.44	0.92	0.74	1.14	0.003
3	rs2215379	0.21	0.21	0.85	1.01	0.89	1.15	0.88	0.18	0.93	0.84	1.03	0.01
4	rs4729938	0.32	0.31	0.33	1.06	0.95	1.18	0.72	0.027	1.11	1.01	1.22	0.08

1 Fisher’s exact test.

2 Odds ratio.

3 Hardy–Weinberg equilibrium p-value in controls.

4 Breslow-Day test p-value.

### Population Stratification Analysis

Using HapMap data, we confirmed that CEU, YRI, CHB, and JPT were divided into three ethnic clusters (Figure S2). Using HapMap data of CEU and YRI and representatives of our confirmation sample from 105 randomly selected cases and controls, we also confirmed three ethnic clusters (Figure S3). There was no obvious population stratification between cases and controls in our confirmation sample set (Figure S4). Our results thus should not be considered a false-positive association that was simply caused by population stratification.

### Cognitive Function Analysis

**Figure 1:**
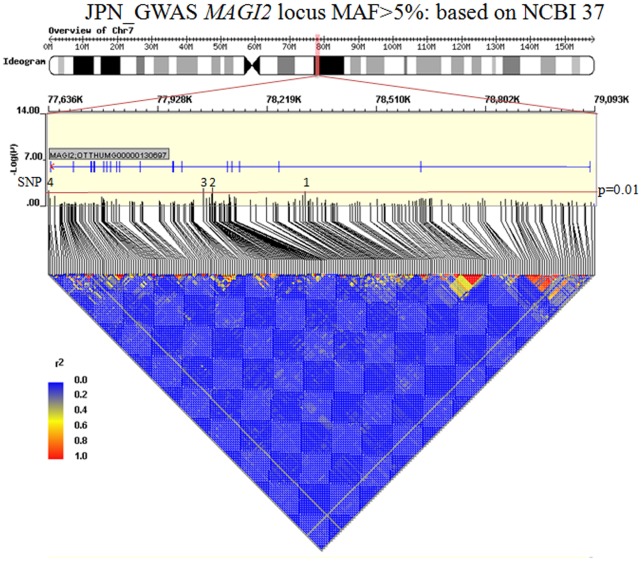
Wisconsin Card Sorting Test composite scores. rs2190665: C allele is risk allele, rs4729938:G allele is risk allele.

We investigated genetic effects of rs2190665 and rs4729938 on WCST. Results are shown in Figure 1. There was no significant difference in clinical information between two groups divided by genotypes of rs2190665 and rs4729938 in cases and controls (Table S1).

In terms of rs2190665, the WCST score was significantly better in the G (protective allele) carrier group (p = 0.034) in cases. In controls, there was no significant cognitive difference between two groups. In terms of rs4729938, the WCST score was worse in the G (risk allele) carrier group in cases, although this value did not reach statistical significance (p = 0.08).

## Discussion

In this study, we investigated the association between 4 SNPs within *MAGI2* and schizophrenia in the Japanese population. We detected suggestive evidence of associations between *MAGI2* and schizophrenia in the joint analysis; however, as the JGWAS dataset was included in the joint analysis, evidence for association might be overestimated. To address this issue, we tested the association between candidate SNPs from our joint analysis and cognitive performance measured by WCST. This analysis was of interest for us as MAGI2 is indirect modulator of NMDA neurotransmission and may impact cognitive function associated with NMDA signaling. Therefore if the two SNPs (rs2190665 and rs4729938) were genuinely associated with schizophrenia, then carriers of risk alleles would likely have deficits in cognitive processing assessed by WCST, as the central executive function is often impaired in schizophrenic patients [19], [20]. All participants in this study were of Japanese descent and recruited from the central area of Japan. We confirmed the lack of stratification within our cases and controls in this study by genotyping and analyzing 98 independent SNPs using our confirmation sample set (Figures S2, S3 and S4). Our association study and cognitive function analysis may be more accurate than research using genomes from people living in the Europe or the United States using the same sample size.

There were several important findings in our study. First, we showed that two SNPs (rs2190665 and rs4729938) may be associated with schizophrenia. Due to the gene length and coarse LD pattern of *MAGI2*, it has been difficult to conduct association studies covering the whole *MAGI2* region without using GWAS results as a screening set. One SNP (rs2190665) is located in intron and the G allele may be a protective allele with an odds ratio of 0.88. The other SNP (rs4729938) is located about 9560 base pairs after *MAGI2,* and the G allele may be a risk allele with an odds ratio of 1.11, and the possibility of false-positive findings due to population stratification seems to be low. Furthermore, we investigated the association between two SNPs and cognitive performance in cases and controls. We could observe significant difference between C/C and G carrier groups of rs2190665 in terms of cognitive performance measured by WCST. The G carrier group (protective allele) in cases scored significantly better than the C/C group. In the control group, there were no significant differences in rs2190665 between the C/C and G carrier groups. This SNP may not be associated with cognitive performance in controls. The G (risk allele) carrier group of rs4729938 in cases had lower WCST scores, although differences did not reach statistical significance. It is possible that if we increase the sample size, we might detect significant difference between C/C group and G carrier groups for rs4729938 in cases. In contrast, we did not find any significant differences between the C/C and G carrier group for rs4729938 in the control group. This SNP may not be associated with cognitive performance in controls.

Several limitations should be considered when interpreting the results of our study. First, in terms of sample size, the replication dataset may not have sufficient statistical power to detect associations between SNPs with low genotype relative risk (GRR) and schizophrenia. In other words, our sample has statistical power greater than 0.8 for the detection of association signals at nominal statistical significance, of the polymorphism with a minor allele frequency of 0.05, when the GRR is 1.30. Therefore, the possibility of association between the schizophrenia and common SNPs with a GRR <1.30 cannot be excluded. Furthermore, the JGWAS may not have sufficient power to detect associations between SNPs with low GRR and schizophrenia. Therefore, other relevant common variants in the *MAGI2* region, which the JGWAS cannot identify, may exist.

The second limitation is that our study design was based on the common disease, common variant hypothesis, based on which we applied a minor allele frequency threshold (>5%) and selected four SNPs for follow up. In the best case scenario, common variants detected in GWAS can explain only part of the heritability in cases of schizophrenia (∼30%) [4] and missense or nonsense mutations on the one side and structural variations (i.e., copy number variants) on the other side are likely to contribute to the increased susceptibility [21]. Recently, the concept of synthetic associations has been suggested, though some objections exist [22]. Uncommon or rare genetic variants can easily create synthetic associations that are credited to common variants. This possibility requires careful consideration in the interpretation and follow up of GWAS signals [23].

The third limitation is that cases and controls in replication samples were not matched in terms of age in our association study. In other words, although highly unlikely, the controls may develop schizophrenia at some point in life, as they were significantly younger than cases.

The fourth limitation is about significant level in our association study. We used JGWAS dataset as a prioritization tool to greatly reduce numbers of candidate SNPs. In joint analysis, three of four association signals with p<0.05 obtained might not survive the statistical significance threshold after multiple comparisons using the Bonferroni correction or the SNPSpD (Supplementary Methods S1) [18]. In addition, the possibility of inflation of p-value of JGWAS results might affect the results in joint analysis, thus the genetic evidence from this study should be regarded as suggestive.

The fifth limitation is about the WCST. The WCST has been used to estimate executive function in schizophrenia, however, several studies have indicated that WCST performance was mainly influenced by individual specific environmental variance rather than genetic variance [24], [25], [26]. Other cognitive tests that are not be susceptible to the environmental influence would be useful for evaluation of the further relationship between *MAGI2* and cognitive function.

In conclusion, common variants in *MAGI2* selected based on JGWAS findings, may be associated with increased schizophrenia risk in a Japanese population. Moreover, we have provided evidence that common SNPs in the *MAGI2* gene region increase risk of cognitive impairment in schizophrenia. The nature of this impairment is consistent with findings from a variety of studies of NMDA-based signaling cascades in excitatory neurotransmission. In particular, executive function became progressively more compromised with increased ‘risk allele load’. Our results thus extend findings for the role for *MAGI2* in serious neuropsychiatric conditions by suggesting that a mutation in *MAGI2* may be present at the level of brain information processing implicated in cognitive impairment.

## Materials and Methods

### Participants

This study was approved by the Ethics Committees of the Nagoya University Graduate School of Medicine and associated institutes and hospitals. Written informed consent was obtained from all participants. In addition, the patients’ capacity to consent was confirmed by the family member when needed. Subjects with legal measure of reduced capacity were excluded. Patients were included in the study if they (1) met DSM-IV criteria for schizophrenia, (2) were physically healthy and (3) had no mood disorders, substance abuse, neurodevelopmental disorders, epilepsy or known mental retardation. A general characterization and psychiatric assessment of subjects is available elsewhere [16]. Controls were selected from the general population. Control subjects had no history of mental disorders, based on questionnaire responses from the subjects themselves during the sample inclusion step, and based on an unstructured diagnostic interview done by an experienced psychiatrist during the blood collection step.

The JGWAS sample was comprised of 575 patients with schizophrenia (43.5±14.8 years (mean±s.d.), male 50%) and 564 healthy controls with no personal or family history of psychiatric illness (44.0±14.4 years (mean±s.d.), male 49.8%). All subjects were unrelated, living in the central area of the Honshu island of Japan and self-identified as members of the Japanese population.

For SNP association analysis, we used an independent Japanese sample set (confirmation sample) comprising 1624 cases (aged 46.5±14.5 years, male 50.6%) and 1621 controls (aged 45.1±14.0 years, male 49.0%). For analysis of cognitive performance, we investigated 114 cases (aged 44.9±13.5 years, male 60.5%) and 91 controls (aged 24.9±6.17 years, male 64.8%).

### SNP Prioritization Step

**Figure 2:**
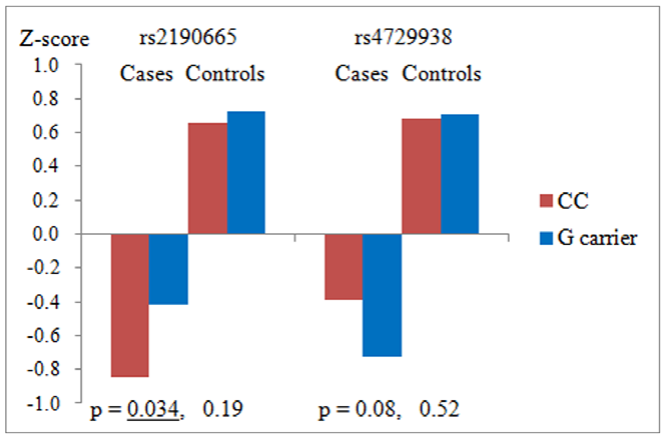
Linkage disequilibrium structure of *MAGI2.* MAF: minor allele frequency, SNP: single nucleotide polymorphism, r^2^: correlation coefficient.

To reduce the number of candidate SNPs for genotyping (due to the length of *MAGI2*) we used the JGWAS dataset as a prioritization tool. All SNPs that were represented on Affymetrix 5.0 array (Affymetrix, Santa Clara, CA) across the *MAGI2* gene and 5% upstream and downstream were selected. A total of 348 SNPs were in this region. Of those, 32 were monomorphic, 80 were excluded due to the low minor allele frequency in controls (<5%), 8 had genotyping call rates <90%, and 7 had significant deviation from Hardy-Weinberg equilibrium (HWE) (p<0.001). 257 SNPs were included in further analyses. The candidate SNPs were defined based on a statistical significance level of p<0.01. Highly correlated markers based on r^2^>0.8 to a more significant marker within 100 kb (r^2^ was based on HapMap information [release 24] and our own GWAS from controls) were then removed. Finally we selected 4 nonredundant SNPs within the *MAGI2* locus (Table 2). Linkage disequilibrium (LD) structure of the MAGI2 region, allelic p values of each SNP in JGWAS, and positions of the four selected SNPs are shown in Figure 2. The LD structure of the four SNPs is shown in Figure S1. All four SNPs were intronic polymorphisms. We investigated the function of these 4 SNPs and other SNPs within the LD of these 4 SNPs, using SNP function prediction (http://snpinfo.niehs.nih.gov/snpfunc.htm) [27]. There were no SNPs with already known functions (Table S2).

**Table 2:** Selected single nucleotide polymorphisms (SNPs) based on results of the Japanese genome-side association study (JGWAS) in the *MAGI2* region.

Number	SNP	Physical Position^1^		LD block	M	m	JGWAS (Cases 575, Controls 564)					
							Cases	Controls	P-value^2^	OR^3^	L95	U95
1	rs2190665	78322159	Intron	1	C	G	0.40	0.46	0.0098	0.80	0.68	0.95
2	rs10260177	78075634	Intron	2	T	C	0.03	0.05	0.0028	0.50	0.32	0.80
3	rs2215379	78050595	Intron	3	C	T	0.18	0.23	0.0037	0.74	0.60	0.91
4	rs4729938	77637379	Intergenic	4	C	G	0.36	0.30	0.0077	1.27	1.07	1.52

1 Ensemble GRCh37 (MAGI2 : from 77646939 to 79082890).

2 Fisher’s exact test.

3 Odds ratio.

### Genotyping and Data Analysis

DNA was extracted from peripheral blood according to standard protocols [28]. Genotyping was performed using a fluorescence-based allelic discrimination assay (Taqman, Applied Biosystems, Foster City, CA). To exclude low-quality DNA samples or genotyping probes, data sets were filtered on the basis of SNP genotype call rates (95% completeness) or deviation from the HWE (p<0.05) in the control sample. Subjects whose percentage of missing genotypes was >10% or who had evidence of possible DNA contamination were excluded from subsequent analyses. All allele-wise association analyses (JGWAS or confirmation sample) were carried out by calculating the p values for each candidate SNP. Significance was determined at the 0.05 level using Fisher’s exact test. All p values were two-sided. To reduce the total number of tests, clearly unassociated markers were removed in the first stage involving the screening sample of the present study. Next, conditional on the findings of the first stage, which used a less stringent nominal level, was tested in the second stage involving the confirmation sample using the augmented data and data from the first stage. In this joint analysis, p values were generated by Cochran-Mantel-Haenszel stratified analysis, and the Breslow-Day test was performed for evaluation of heterogeneous associations as implemented in PLINK v1.07 [29]. Statistical significance was set at nominal level (p<0.05) in association study using confirmation sample set and joint analysis. The SNPSpD can make a simple correction for multiple testing of SNPs in LD with each other, on the basis of the spectral decomposition (SpD) of matrices of pairwise LD between SNPs [18]. This method is less conservative than the Bonferroni correction. Using the program (http://gump.qimr.edu.au/general/daleN/SNPSpD/), we calculated the adjusted p-value in our total sample set (n = 4353) including all cases and controls.

### Population Stratification Analysis

To avoid false-positive associations caused by potential population stratification, we performed population stratification analysis on our confirmation sample set, using genotype data on 98 additional random LD independent SNPs. We obtained data on these 98 SNPs from 638 HapMap samples (174 CEU [Utah residents with ancestry from northern and western Europe in the United States], 209 YRI [Yoruba in Ibadan, Nigeria], 139 CHB [Han Chinese in Beijing], and 116 JPT [Japanese in Tokyo]) from HapMap public release 28 at http://hapmap.ncbi.nlm.nih.gov. First we investigated whether these SNPs worked well to differentiate HapMap sample sets into distinct clusters according to their ethnicity. Second we investigated whether these SNPs worked well to differentiate confirmation sample set from non Japanese sample set (HapMap dataset). Third we investigated uniformity of the distribution of cases and controls from confirmation sample within the population stratification plot using Structure version 2.3.3 software (http://pritch.bsd.uchicago.edu/structure.html). We applied an admixture model and an independent allelic frequency model and ran at K = 3, where K is the number of potential classifications of tested samples. For all Structure runs, we set the parameters with a burn-in of 10 000 iterations and 20 000 follow-on iterations.

### Neurocognitive Assessment

The Wisconsin Card Sorting Test (WCST) scores were used in this study because they (1) are known to be sensitive to the effects of glutamate manipulations [30] and (2) are important predictors of clinical outcome [31].

The WCST [32] mainly assesses executive function including cognitive flexibility in response to feedback. We used a modified and computerized version of the test: Wisconsin Card Sorting Test (Keio Version) (KWCST) [33]. The outcome measures commonly used in KWCST were numbers of categories achieved (CA), total errors (TE) and perseverative errors of Milner (PEM) and Nelson types (PEN) [34]. We selected CA and PEN as outcomes in the current study because TE, PEM and PEN are highly correlated. CA is the number of categories for which six consecutive correct responses are achieved (eight is the maximum number of categories that can be achieved), and is the sum measure of the level of conceptual shifts in the KWCST. PEN is the number of incorrect responses in the same category as the immediately preceding incorrect response (maximum of 47 perseverative errors) [35], [36].

### Clinical Information

Patients’ records were used to obtain relevant clinical information (e.g. age, education, Chlorpromazine (CPZ) equivalent doses, age at onset and duration of illness).

CPZ equivalent doses of antipsychotic medications were calculated based on the report by Inagaki et al. [37], [38]. Medication status of patients was investigated on the day when cognitive tests were conducted. Patients’ medication status and positive and negative symptom scale (PANSS) [39] scores were obtained at the time of cognitive assessment.

### Analysis of Cognitive Performance

We checked the effect of two SNPs on cognitive performance measured by WCST within 114 schizophrenic patients and 91 healthy controls. To avoid the problem of multiple comparisons, WCST CA and PEN scores were used to synthesize the WCST composite score, and the first factor scores were derived from an unrotated principal component analysis in all participants. Several studies investigating association study and cognitive tests have been conducted [40], [41]. In these studies, unrotated principal component analysis was used to calculate general cognitive ability of subjects from many cognitive measures. In the current study, calculated WCST composite scores were converted to Z-scores using the standardized mean = 0 and standard deviation = 1 from the data set of all participants, such that lower values reflect worse performance. The first unrotated factor explained 89.7% of the variance.

We compared Z-scores of WCST composite scores between two groups divided by genotype, CC and G carrier groups, in cases and controls using the student t-test. Clinical information (age, education, CPZ equivalent doses, age at onset, duration of illness and PANSS) of two groups divided by genotype between schizophrenia cases and control subjects was compared using the student t-test and Welch’s t-test. We compared sex between cases and controls using Fisher’s exact test. IBM SPSS statistical software, version 19 (IBM Japan, Tokyo, Japan) was used for all analyses. All tests were two-tailed and significance was set at p = 0.05.

## Supporting Information

Figure S1 Linkage disequilibrium structure of four selected single nucleotide polymorphisms (SNPs).
(JPG)

Figure S2 Population stratification analysis within HapMap samples.
(JPG)

Figure S3 Population stratification analysis within HapMap samples and randomly selected cases and controls.
(JPG)

Figure S4 Population stratification analysis within our cases and controls.
(JPG)

Table S1 Clinical information and Wisconsin Card Sorting Test composite scores.
(XLS)

Table S2 SNP function prediction in the 4 SNPs and other SNPs within LD of the 4 SNPs.
(XLSX)

Methods S1 Multiple comparison.
(DOC)
