# Correction: Common Variants in *MAGI2* Gene Are Associated with Increased Risk for Cognitive Impairment in Schizophrenic Patients

**DOI:** 10.1371/annotation/47ca9c23-9fdd-47f6-9d36-db0a31769f22

**Published:** 2012-09-18

**Authors:** Takayoshi Koide, Masahiro Banno, Branko Aleksic, Saori Yamashita, Tsutomu Kikuchi, Kunihiro Kohmura, Yasunori Adachi, Naoko Kawano, Itaru Kushima, Yukako Nakamura, Takashi Okada, Masashi Ikeda, Kazutaka Ohi, Yuka Yasuda, Ryota Hashimoto, Toshiya Inada, Hiroshi Ujike, Tetsuya Iidaka, Michio Suzuki, Masatoshi Takeda, Nakao Iwata, Norio Ozaki

The images for Figures 1 and 2 were incorrectly switched. The image that appears as Figure 1 should be Figure 2, and the image that appears as Figure 2 should be Figure 1. The figure legends appear in the correct order.

