# Supplementary figures and images for: Common Variants in MAGI2 Gene Are Associated with Increased Risk for Cognitive Impairment in Schizophrenic Patients
Source: PLoS One. 2012 May 23;7(5):e36836. doi: 10.1371/journal.pone.0036836 (PMC3359314; doi:10.1371/journal.pone.0036836)

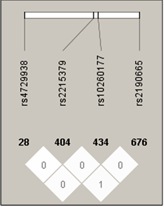

Supplement: Figure S1 — Linkage disequilibrium structure of four selected single nucleotide polymorphisms (SNPs). (JPG) [file pone.0036836.s001.jpg]

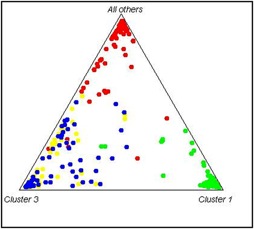

Supplement: Figure S2 — Population stratification analysis within HapMap samples. (JPG) [file pone.0036836.s002.jpg]

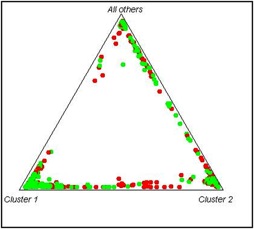

Supplement: Figure S4 — Population stratification analysis within our cases and controls. (JPG) [file pone.0036836.s004.jpg]
