# Supplementary material for: Common Variants in MAGI2 Gene Are Associated with Increased Risk for Cognitive Impairment in Schizophrenic Patients
Source: PLoS One. 2012 May 23;7(5):e36836. doi: 10.1371/journal.pone.0036836 (PMC3359314; doi:10.1371/journal.pone.0036836)
Supplement: Methods S1 — Multiple comparison. (DOC) [file pone.0036836.s007.doc]

Method S1. Multiple comparison

**Threshold calculated by SNPSpD (http://gump.qimr.edu.au/general/daleN/SNPSpD/)**

Single Nucleotide Polymorphism Spectral Decomposition (SNPSpD) - RESULTS

Matrix of pairwise LD correlations for your markers:

1 2 3 4

1 1 0.05 0.01 -0.00

2 0.05 1 0.01 0.04

3 0.01 0.01 1 -0.02

4 -0.00 0.04 -0.02 1

Original (total) number of marker loci (M) after removing redundant (collinear) SNPs:

4

For factor 1 to M, original eigenvalues associated with the LD correlation matrix:

1 1.0691

2 1.0005

3 0.9905

4 0.9399

Variance of the observed eigenvalues:

0.0028

Effective number of independent marker loci [Meff]:

3.9979

Experiment-wide significance threshold required to keep Type I error rate at 5%:

0.0125066270171466
